# Causal association between dietary factors and esophageal diseases: A Mendelian randomization study

**DOI:** 10.1371/journal.pone.0292113

**Published:** 2023-11-29

**Authors:** Menglong Zou, Qiaoli Liang, Wei Zhang, Ying Zhu, Yin Xu

**Affiliations:** 1 The First Hospital of Hunan University of Chinese Medicine, Changsha, Hunan, China; 2 Graduate School of Hunan University of Chinese Medicine, Changsha, Hunan, China; 3 Zhuhai Second Hospital of Chinese Medicine, Zhuhai, Guangdong, China; University of Central Florida College of Medicine, UNITED STATES

## Abstract

**Background:**

Using Mendelian randomization (MR) approach, our objective was to determine whether there was a causal association between dietary factors and gastroesophageal reflux disease (GERD), Barrett’s esophagus (BE), or esophageal cancer (EC).

**Methods:**

Genome-wide association study (GWAS) data for eighteen types of dietary intake were obtained from the UK Biobank. GWAS data for GERD, BE, and EC were sourced from the FinnGen consortium. We performed univariable and multivariable MR analysis to assess the cause effect between dietary factors and esophageal diseases. MR results were expressed as odds ratios (OR) with 95% confidence intervals (CI).

**Results:**

Raw vegetable intake was associated with a lower risk of GERD (OR = 0.478; *P* = 0.011). On the contrary, cooked vegetable intake increased the risk of GERD (OR = 1.911; *P* = 0.024). Bread intake was associated with increased odds of BE (OR = 6.754; *P* = 0.007), while processed meat intake was associated with reduced risk of BE (OR = 0.210; *P* = 0.035). We also observed evidence that increased consumption of dried fruit (OR = 0.087; *P* = 0.022) and salt added to food (OR = 0.346; *P* = 0.045) could prevent EC. The results of multivariable MR showed that the protective effect of consumption of salt added to food on EC was no longer significant after adjusting for the consumption of dried fruit.

**Conclusion:**

Vegetable consumption was associated with GERD, whereas consumption of bread and processed meat was associated with BE. Dried fruit intake was associated with a lower risk of EC, and the protective effect of consumption of salt added food on EC may also be mediated by consumption of dried fruit. Future research should be performed to investigate the mechanisms behind these cause-and-effect relationships to reduce the burden of disease caused by dietary habits.

## Introduction

Esophageal cancer (EC) is one of the most commonly diagnosed malignancies worldwide and ranks sixth in cancer-related mortality [1]. Estimates predicted a total of 604,100 new cases and 544,076 deaths from EC in 2020 alone [2]. Esophageal adenocarcinoma (EAC) is increasing rapidly and has become the predominant subtype of EC in European populations [3]. Barrett’s esophagus (BE) refers to the metaplastic alteration of the esophageal mucosa from a normal squamous epithelium to a columnar epithelium, which is recognized as a precursor lesion for the development of EAC [4, 5]. The development of EAC is typically characterized by a sequential progression from BE metaplasia to dysplasia, culminating in the emergence of invasive carcinoma. Approximately 10–15% of patients suffering from gastroesophageal reflux disease (GERD) will experience the development of BE, which is caused by a metaplastic transformation resulting from chronic mucosal injury due to repeated episodes of acid reflux [6]. With the worldwide increase in the incidence of EAC in recent decades [7], it is important to identify modifiable risk factors that may contribute to the development of GERD.

Research has been carried out since the twentieth century to determine the risk factors for esophageal diseases. The association between dietary factors and esophageal diseases has been investigated in different parts of the world, but with different and sometimes contradictory findings. Alcohol, for example, may have a direct toxic effect on the esophageal mucosa and reduces lower esophageal sphincter (LES) pressure, especially when consumed in large quantities [8]. However, epidemiological research on alcohol intake and esophageal diseases has shown contradictory results [9–12]. Most physiological research has not been able to determine the role of diet (especially fat) in GERD [13]. Some researchers even advocate a low-fat diet for people with GERD, but this is inappropriate [14]. Conversely, a number of previous researchers have demonstrated an association between fat and GERD [15]. In conclusion, the association between dietary factors and esophageal diseases has attracted considerable attention. However, establishing a link between dietary and esophageal diseases has been a challenge since clinical case-control studies are susceptible to bias from confounders, such as reverse causality.

Randomized controlled trials (RCTs) are considered the gold standard for the establishment of causality. However, RCTs are not always practical as they can be excessively costly and even unethical. RCTs cannot provide answers to many causal questions, such as the long-term consequences of using addictive substances or those of potentially harmful substances. The instrumental variables approach is another statistical method used to investigate the causality of associations between exposure and outcome. In fact, the concept of the instrumental variables approach was first introduced by econometricians about a century ago and later applied to medical statistics [16, 17]. Mendelian randomization (MR) is a novel method of epidemiological research that uses genetic variants as instrumental variables to assess the presence or absence of causal effects between exposure and outcome [18–20]. Evidence of causal effects analyzed in this method greatly reduces the bias caused by confounders in observational studies, because the genetic variants are randomly assigned at the time of conception [21]. Genetic analysis of traits with modest heritability (e.g., diet) is now possible in large-scale biobanks. A genome-wide association analysis of 85 dietary patterns based on the UK Biobank food frequency questionnaire by Cole et al. provides a deeper exploration of the causal relationship between diet and disease [22]. In this study, we used MR approach to evaluate the causal effect of eighteen dietary patterns of genetic susceptibility on the risk of GERD, BE, and EC, providing an indication for primary disease prevention.

## Materials and methods

### Study design

**Fig 1:**
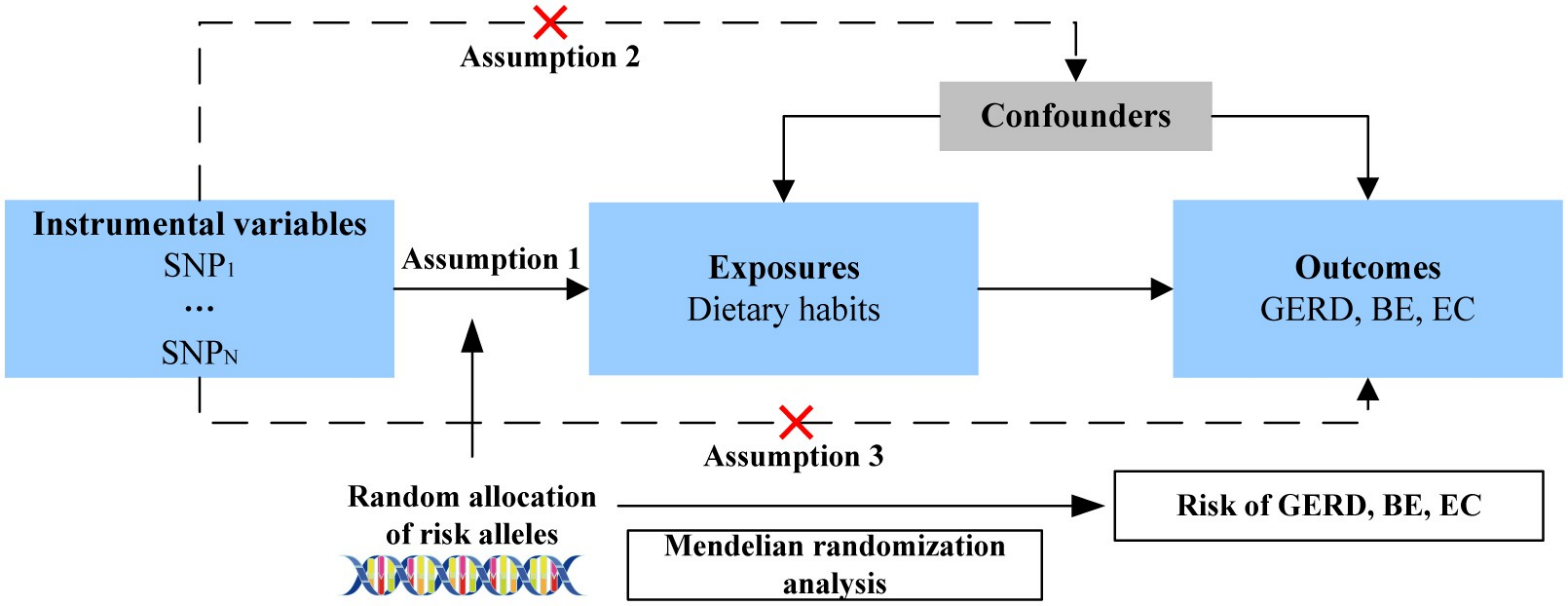
Flowchart describing research process.

A two-sample MR analysis was used to investigate the causal relationship between 18 dietary habits and esophageal disease (Fig 1). Representative phenotypic single-nucleotide polymorphisms (SNPs) were selected as instrumental variables [23]. The instrumental variables for phenotypes should meet the three main assumptions: (1) SNPs should be strongly associated with corresponding phenotype; (2) SNPs should be unaffected by potential confounders of the exposure-outcome association; (3) direct links between SNPs and outcome are not available [24]. The data used in this study were obtained from public databases that had already been ethically approved for the original study. Therefore, no additional ethical approval was required for this study.

### Data source

Eighteen exposure factors related to dietary habits were included in this study, including beverage intake (alcohol, coffee, and tea), staple food intake (cereal and bread), fruit intake (fresh fruit and dried fruit), vegetable intake (cooked vegetable and raw vegetable), meat intake (beef, pork, lamb, processed meat, poultry, oily fish, and non-oily fish), salt added to food, and another food intake (cheese). These GWAS summary data were obtained directly or indirectly from the UK Biobank (http://www.nealelab.is/uk-biobank) by the integrative epidemiology unit (IEU) open GWAS project (https://gwas.mrcieu.ac.uk/) [25, 26]. Information on the 18 dietary patterns was collected retrospectively using a dietary frequency questionnaire, which is publicly available in the UK Biobank (https://biobank.ctsu.ox.ac.uk/crystal/label.cgi?id=100052). Questionnaire submission was rejected if participants’ answers were unrealistic. Details of the 18 dietary questionnaires are given in S1 Table (S1 Appendix). The GWAS summary data for esophageal diseases were extracted from the FinnGen consortium R8 release (https://www.finngen.fi/en) [27], which includes 22,867 cases and 292,256 controls for GERD, 946 cases and 292,256 controls for BE, and 503 cases and 259,583 non-cancer controls for EC. There was little overlap between the exposure and outcome samples.

### Genetic instrumental variables selection

It is known that *P* < 5×10^−8^ is the threshold for genome-wide significance. Therefore, we extracted SNPs strongly associated with each dietary pattern based on a threshold of *P* < 5×10^−8^. Linkage disequilibrium (LD) clumping was performed using parameters (r2 < 0.001 and window size = 10,000 kb) to ensure the independence of these SNPs. In addition, the *F* statistic was calculated to assess the bias of the weak instrumental variables [28]. When the *F* statistic > 10, the bias of weak instrumental variables can be ignored [28]. Data on exposure and outcome were merged and harmonized by effect alleles [29]. We discarded SNPs associated to outcomes from the merged data [30].

### Heterogeneity and pleiotropy analysis

Cochran’s Q test was performed to evaluate heterogeneity and a significant *P* value indicates the presence of heterogeneity [31, 32]. MR Egger intercept analysis was used to assess horizontal pleiotropy [33]. In the presence of pleiotropy (*P* < 0.05), we used MR-PRESSO to identify potential outliers [23]. The MR analysis was re-conducted after removing the outliers.

### Univariable and multivariable MR analysis

The inverse variance weighting (IVW) method was used as the primary analysis to assess the causal effect of dietary habits on the risk of esophageal diseases. Two additional MR methods (MR Egger and weighted median) were used as a complement. We used multivariable MR analysis to adjust for potential confounders between different dietary patterns. MR results were expressed as odds ratios (OR) with 95% confidence intervals (CI). A bilateral *P* value < 0.05 was considered statistically significant. In this study, all analysis were performed using the “TwoSampleMR (version 0.5.6)” package in the R software (version 4.2.2).

## Results

### Instrumental variables for dietary factors

Overall, eighteen diet-related GWAS data were included in our study for analysis (Table 1). The amounts of SNPs for each dietary pattern ranged from 8 to 106. Detailed information about these SNPs is presented in S2 Table (S1 Appendix). The F statistics are all greater than 10, indicating that the bias of weak instrumental variables on the results of this study can be ignored.

**Table 1:** Summary of eighteen dietary habits.

GWAS id	Exposure	Number of SNPs	Sample	*R*^*2*^ (%)	*F*
ukb-a-25	Alcohol intake frequency	44	336965	0.78	59.85
ukb-b-11348	Bread intake	32	452236	0.30	41.93
ukb-b-1489	Cheese intake	65	460006	0.56	39.23
ukb-b-15926	Cereal intake	43	451486	0.44	45.22
ukb-b-16576	Dried fruit intake	43	441640	0.43	42.07
ukb-b-3881	Fresh fruit intake	55	421764	0.57	46.20
ukb-b-17627	Non-oily fish intake	11	460880	0.11	44.84
ukb-b-2209	Oily fish intake	63	435435	0.61	45.18
ukb-b-1996	Raw vegetable intake	22	460443	0.19	38.40
ukb-b-8089	Cooked vegetable intake	17	461053	0.14	37.63
ukb-b-2862	Beef intake	17	446462	0.15	41.53
ukb-b-5640	Pork intake	14	428860	0.11	37.72
ukb-b-14179	Lamb intake	32	460162	0.28	39.70
ukb-b-6324	Processed meat intake	23	447485	0.19	38.60
ukb-b-8006	Poultry intake	8	461981	0.06	32.55
ukb-b-5237	Coffee intake	40	461900	0.68	73.15
ukb-b-6066	Tea intake	41	448651	0.56	61.12
ukb-b-8121	Salt added to food	106	462630	1.15	50.77

GWAS, Genome-Wide Association Studies; SNPs, Single-nucleotide polymorphisms; F, F statistics; R^2^, phenotype variance explained by genetics.

### Univariable MR analysis of esophageal diseases

**Fig 2:**
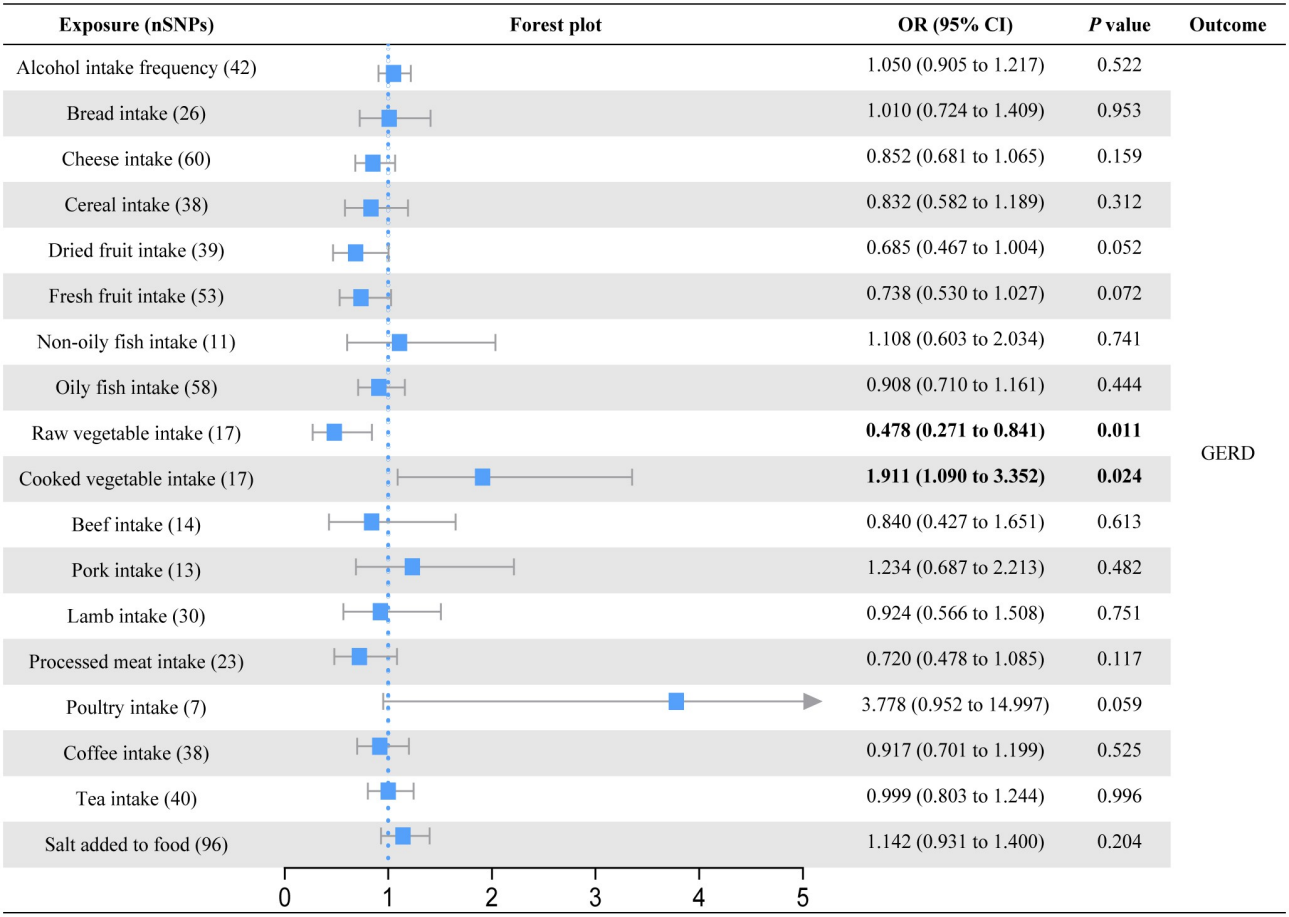
Forest plot of the causal effect of dietary habits on GERD using the inverse variance-weighted method.

**Fig 3:**
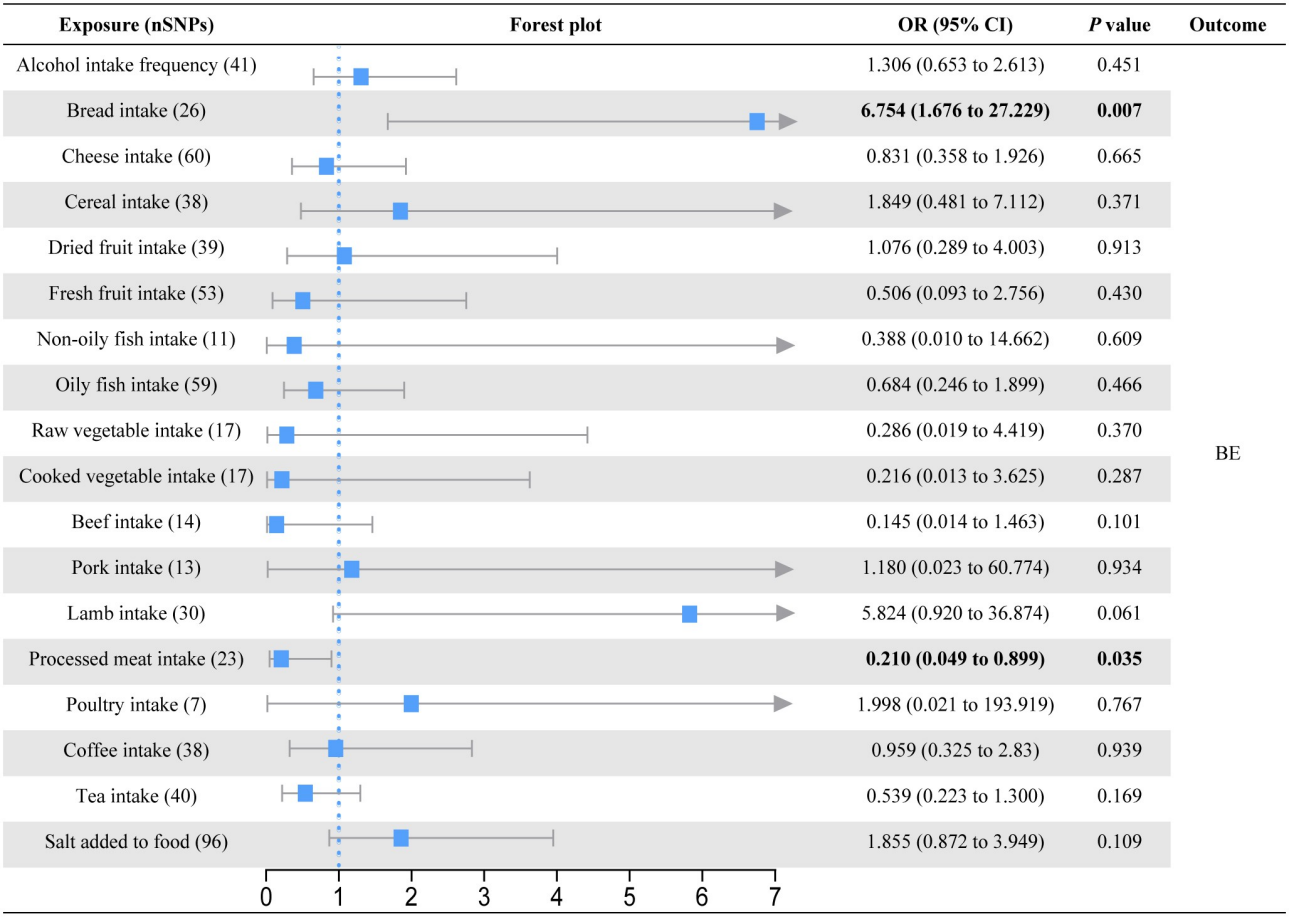
Forest plot of the causal effect of dietary habits on BE using the inverse variance-weighted method.

**Fig 4:**
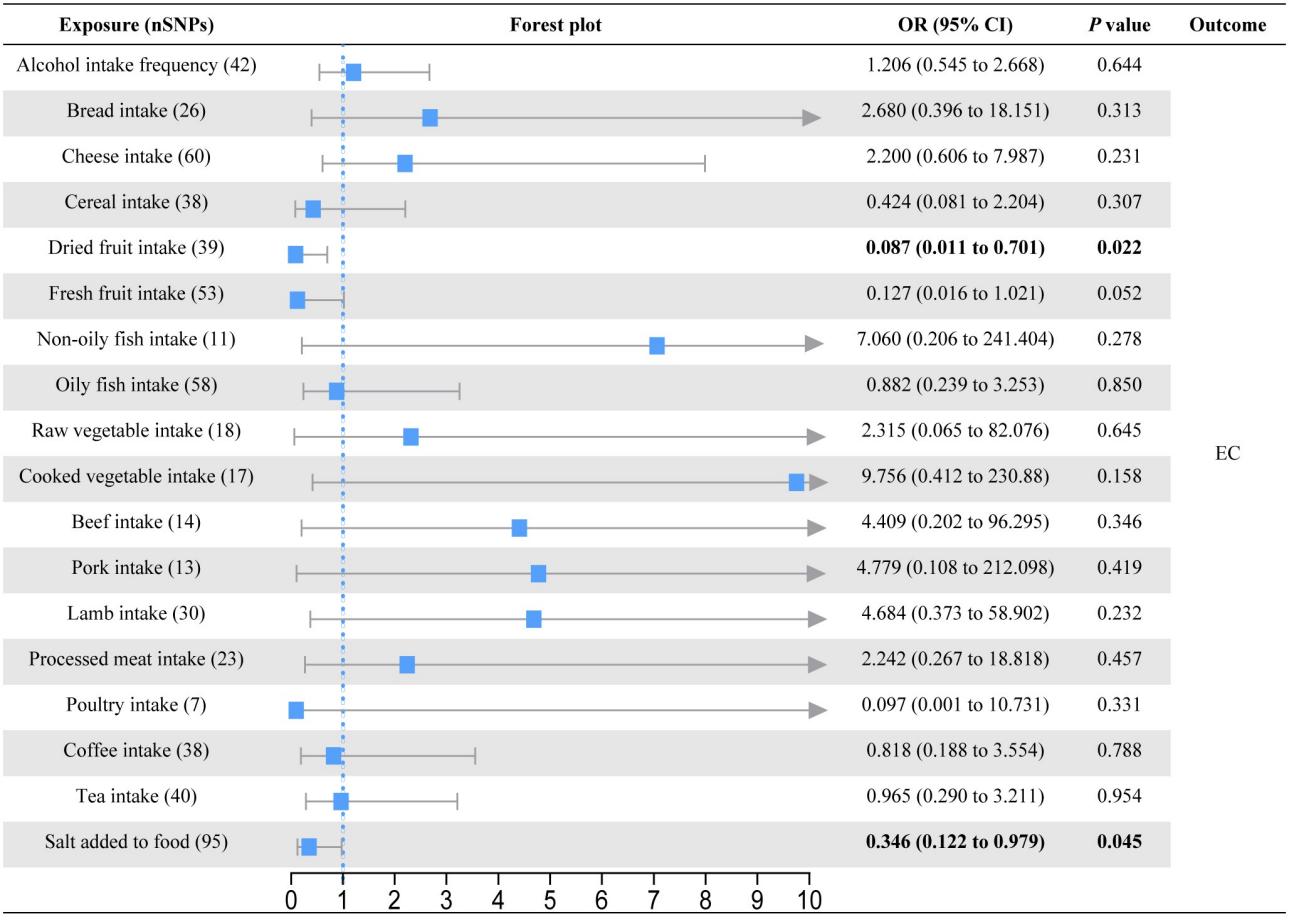
Forest plot of the causal effect of dietary habits on EC using the inverse variance-weighted method.

In the primary univariable MR analysis, six causal associations from eighteen dietary patterns to esophageal diseases were identified (Figs 2–4; S3-S5 Tables in S1 Appendix). Raw vegetable intake was associated with a lower risk of GERD (OR = 0.478; 95% CI = 0.271 to 0.841; *P* = 0.011) but not with BE (OR = 0.286; 95% CI = 0.019 to 4.419; *P* = 0.370) and EC (OR = 2.315; 95% CI = 0.065 to 82.076; *P* = 0.645). On the contrary, cooked vegetable intake levels increased the risk of GERD (OR = 1.911; 95% CI = 1.090 to 3.352; *P* = 0.024). Although the results of the MR Egger intercept analysis suggested possible pleiotropy (*P* = 0.027), the MR-PRESSO analysis found no outliers (*P*_global test_ = 0.182) and the Cochran’s Q test found no heterogeneity (*P* = 0.172). Bread intake was strongly associated with an increased risk of BE (OR = 6.754; 95% CI = 1.676 to 27.229; *P* = 0.007). Processed meat intake reduced the risk of BE (OR = 0.210; 95% CI = 0.049 to 0.899; *P* = 0.035). We also observed evidence that consumption of dried fruit (OR = 0.087; 95% CI = 0.011 to 0.701; *P* = 0.022) and salt added to food (OR = 0.346; 95% CI = 0.122 to 0.979; *P* = 0.045) could prevent EC. Based on the results of the IVW-MR analysis, there are some critical values that we should be of concern. For the GERD, consumption of dried fruit (OR = 0.685; *P* = 0.052) and fresh fruit (OR = 0.738; *P* = 0.072) tended to decrease disease risk, whereas consumption of poultry tended to increase disease risk (OR = 3.778; P = 0.059). For the BE, lamb intake had a tendency to reduce disease risk (OR = 5.824; *P* = 0.061). For the EC, there was a trend for fresh fruit intake to decrease disease risk (OR = 0.127; *P* = 0.052). In addition to the causal associations identified by the IVW method described above, weighted median method also identified several indicative results that showed trends consistent with IVW method. Based on weighted median method, dried fruit intake (OR = 0.549; *P* = 0.008) was suggested to be associated with a decreased risk of GERD, cooked vegetable intake (OR = 0.023; *P* = 0.030) and beef intake (OR = 0.041; *P* = 0.040) were suggested to be associated with a decreased risk of BE, lamb intake (OR = 18.188; *P* = 0.028) was suggested to be associated with an increased risk of BE, and non-oily fish intake (OR = 123.033; *P* = 0.039) was suggested to be associated with an increased risk of EC (S3-S5 Tables in S1 Appendix).

### Multivariable MR analysis of esophageal diseases

**Fig 5:**
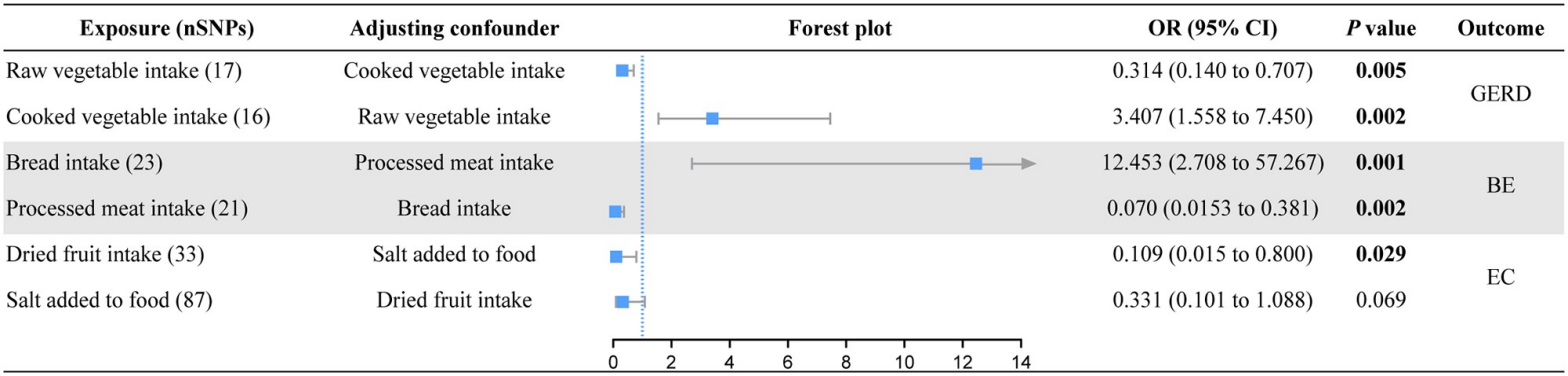
Forest plot of the results of multivariable MR.

Although univariable MR analysis identified causal associations between dietary habits and esophageal diseases, dietary habit may not be a single element. Therefore, we performed a multivariable MR analysis based on the causal associations identified by the IVW method described above (Fig 5; S6 Table in S1 Appendix). The association between raw vegetable intake (adjusted for cooked vegetable intake: OR = 0.314; *P* = 0.005) or cooked vegetable intake (adjusted for raw vegetable intake: OR = 3.407; *P* = 0.002) and GERD remained significant in multivariate MR analysis. Bread intake (adjusted for processed meat intake: OR = 12.453; *P* = 0.001) or processed meat intake (adjusted for bread intake: OR = 0.070; *P* = 0.002) also had similar significant causal effects on BE. Higher dried fruit intake levels also were a protective factor against EC (adjusted for the consumption of salt added to food: OR = 0.109; *P* = 0.029). However, the protective effects of consumption of salt added to food was no longer statistically significant after adjusting for dried fruit intake (adjusted OR = 0.331; *P* = 0.069).

## Discussion

In light of the rapid surge in incidence rates of esophageal diseases over recent decades, pinpointing modifiable risk factors, such as dietary habits, represents an appealing avenue to thwart the initiation and progression of these conditions. In this study, MR analysis was conducted to investigate the potential causal relationship between dietary intake and three different types of esophageal diseases. Using large GWAS data on dietary intake, GERD, BE, and EC, we identified six causal associations, including intake of raw and cooked vegetables with GERD, intake of bread and processed meat with BE, and intake of dried fruit and salt added to food with EC.

Alcohol is one of the most concerning dietary intakes for esophageal diseases. Alcohol consumption has the potential to increase reflux symptoms, damage the esophageal mucosa and even promote carcinogenesis [34]. A small study in 25 healthy volunteers performed by Hamoui et al. showed that the consumption of alcohol reduced LES pressure, which led to an increase in reflux episodes [35]. This is inconsistent with the results of large cohort studies, for example, a case-controlled assessment of 3,153 people with GERD compared with 40,210 people without GERD by Nilsson et al. showed no association [36]. The two main types of EC are squamous cell carcinoma (ESCC) and EAC. Patients with EAC had a higher total alcohol intake compared to controls [37–39]. However, some researchers have also reported moderate alcohol consumption as a protective factor for EAC [40, 41], whereas some studies found no effect [42–45]. Alcohol consumption has been shown to be a risk factor for ESCC in most studies [39, 43, 46]. Again, a number of studies found an association between lifetime alcohol consumption and a lower risk of BE [45, 47], but some reports found no association [48–52]. According to Kubo et al, an increased risk mediated by alcohol consumption was only found when BE cases were compared with GERD controls [53]. Similar to alcohol, some studies reported that tea or coffee significantly reduced LES pressure and lower esophageal pH [54, 55]. In this MR analysis, we found no evidence for a causal association between the consumption of alcohol, coffee, or tea and GERD, BE, or EC. Differences in results between studies may be due to the heterogeneity of the cases or controls studied in many trials, as controls from the general population sampled may have undiagnosed GERD or BE. In addition, observational or retrospective studies may be subject to unavoidable confounders that interfere with the estimation of exposure-outcome correlations and weaken the power of the findings to make precise causal decisions. This means that a direct causal relationship could not be proven, although one observational or retrospective study reported a potentially strong association. The MR analysis approach largely avoids the interference of potential confounders by introducing genetic variants, thereby providing a relatively precise estimate of causal associations.

Fruit and vegetables are the main sources of antioxidants in the diet. The most protective was dark-green (mustard, spinach) and red-orange (tomatoes, carrots) vegetables, apples, berry juices, and citrus fruits [56]. Antioxidants provide protection by interacting with reactive oxygen species to attenuate oxidation/nitrosation reactions caused by the overproduction or overaccumulation of these highly reactive molecules. Oxidative/nitrosative stress has been implicated in the development of many human diseases, including EC [57–59]. In patients with BE, GERD-induced esophageal inflammation can lead to the production of free radicals, which promote carcinogenesis by directly damaging DNA and inhibiting apoptosis [60]. A case-control study by Terry et al found that higher dietary intake of antioxidants (β-carotene and vitamin C) was associated with a lower risk of EAC, particularly in patients with GERD, who are thought to have higher levels of oxidative stress [61]. It was found that a higher intake of vegetables was more effective than fruit, which could be explained by the low sugar and energy content [62]. In contrast, an investigation carried out by Zheng et al. [63] did not establish a significant association between the consumption of fruit or vegetables and the occurrence of GERD. Another study conducted by Jarosz and Taraszewska [64] similarly failed to demonstrate any statistically significant associations between fruit intake and GERD (OR = 1.04, *P* = 0.930). No evidence of protection against EC was found in the systematic review performed by Bjelakovic et al. [65–67]. In addition, they found that vitamin A and β-carotene supplementation appeared to be associated with an increased risk of EC. In our study, the weighted median-based approach suggested that dried fruit intake may be associated with a reduced risk of GERD; whereas the IVW-based approach suggested that dried fruit intake was associated with a reduced risk of EC, but the latter may be influenced by salt-added food intake. We found significant effects of vegetable intake (both raw and cooked) on GERD, but part of the causal association was no longer significant after adjusting for confounders. Interestingly, raw vegetable intake was a protective factor for GERD, while cooked vegetable intake was a risk factor. The reason for these differences may be that cooking reduces the antioxidant capacity of vegetables [68]. A study performed by Gunathilake et al. also showed that the total antioxidant capacity of the steamed leaves of *O*.*zeylanica* was significantly lower compared with its raw leaves [69]. Based on the weighted median approach, the consumption of cooked vegetables was suggested to reduce the risk of BE, while raw vegetables were not. There was no evidence in our study that the consumption of vegetables was associated with EC.

In the European prospective investigation, a positive but not statistically significant association between total intake of processed meat or red meat and EAC was found [70]. Kubo and colleagues reported that meat consumption was associated with an increased risk of BE compared with population controls, but among patients with GERD, this was not a risk factor for BE [71]. A case-control study in the USA found a higher risk of EAC with a high intake of meat in the diet (especially red meat) [72]. Gallus and La Vecchia [73], Castellsagué et al. [74] also reported a positive association between high risk of EAC and consumption of processed or red meat, and a negative association with consumption of fish and white meat. In contrast, a high intake of fat or red meat reduced the risk of EAC, according to Wu et al. [75]. High-fat foods release large amounts of cholecystokinin, which delays gastric emptying by stimulating vagal afferent fibers and predisposes to gastro-esophageal reflux [76]. However, in a double-blind randomized controlled trial, low-fat meals with isocaloric delivery had no effect on the number of reflux episodes and mean LES pressure compared to high-fat meals [14]. This contrasts with previous studies that reported changes in the LES pressure and duration of esophageal acid exposure after fat ingestion [77, 78]. Our study found no association between meat consumption (including beef, pork, lamb, processed meat and poultry) and GERD or EC, but did find that eating processed meat reduced the risk of BE. Ganesh et al. reported a 20% reduction in the incidence of EC with increased consumption of fresh fish in a case-control study conducted in Mumbai [79]. However, we did not find a causal relationship between genetically predicted fish intake (oily and non-oily) and GERD, BE or EC based on IVW-MR analysis.

Cereals and legumes are the groups of plants with the highest nutritional requirements in the world. An earlier retrospective cohort study by Yu et al. in Linxian, an area of China with a high prevalence of EC, reported a positive association between increased cereal intake and EC risk [80]. A HELGA cohort study by Skeie et al, consisting of three sub-cohorts from Norway, Sweden and Denmark, reported a negative association [81]. A possible reason for the discrepancy in results is that earlier studies did not take into account the type of cereal. Our MR results did not support a causal association between cereal intake and GERD, BE or EC. An observational study in the USA found no effect of dairy consumption on GERD symptoms [82], while another randomized controlled trial also reported no association between the risk of GERD and the consumption of dairy products (both reduced-fat and full-fat cheeses) [83]. These findings are consistent with our MR results, where we did not find a causal relationship between genetic susceptibility to cheese consumption and GERD, BE or EC. However, we found that bread intake significantly increased the risk of BE, independent of GERD or BE. In addition, we also observed an intriguing finding that increasing the consumption of salt added to food could reduce the risk of EC. After performing multivariable MR analysis, we found that the protective effect of salt-added food intake on EC may be mediated by dried fruit intake (adjusted for the consumption of salt added to food: OR = 0.109; *P* = 0.029; adjusted for the consumption of dried fruit intake: OR = 0.331; *P* = 0.069). Therefore, some caution is needed in interpreting the result that salt added to food may act as a protective factor for EC.

The present study has three strengths. Firstly, to our knowledge, this is the first report to use large-scale GWAS data for a two-sample MR approach to assess the causal association between food intake factors and esophageal diseases. Compared to previous observational and retrospective studies, this approach is less susceptible to the influence of confounders. Secondly, our study reports a causal link between a variety of dietary habits and three types of esophageal disease, some of which have not been reported in previous studies. The rigorous selection of instrumental variables (*P* < 5×10^−8^ and *F* > 10) and the large sample size with little overlap ensure the reliability of our results. Thirdly, multivariable MR analysis assessed and adjusted for potential confounders.

Our study also has limitations. First, MR results are subject to bias due to pleiotropy, which is currently a challenge for all MR analyses. In this study, most of our results were stable. Second, the strength of the evidence provided by the MR results depends largely on the plausibility of the instrumental variable assumptions, and acquired development may buffer the representativeness of the instrumental variables. Third, although we investigated the causal relationship between dietary intake and three esophageal diseases, we did not perform analysis of the subtypes of esophageal diseases. For example, EAC and ESCC are the two main subtypes of EA, and there are differences in the risk factors for these two subtypes. Therefore, further MR analysis using dietary intake as the exposure and disease subtype as the outcome would provide informative results. Unfortunately, there is no large GWAS summary data available for EAC and ESCC. The present study presents findings on the causal association between dietary intake and esophageal disease, which will facilitate future subtype analysis.

## Conclusion

In conclusion, we thoroughly examined the potential causal association between dietary habits and esophageal diseases. Consumption of raw vegetable reduced the risk of GERD, while the opposite was true for cooked vegetable. Bread intake was positively associated with BE risk, while processed meat intake was negatively associated. Dried fruit intake was associated with a lower risk of EC, and the protective effect of consumption of salt added food on EC may also be mediated by consumption of dried fruit. Future research should be performed to investigate the mechanisms behind these cause-and-effect relationships to reduce the burden of disease caused by dietary habits.

## Supporting information

S1 Appendix S1-S6 Tables are included in file. (XLSX)
